# Novel glucose-responsive of the transparent nanofiber hydrogel patches as a wearable biosensor via electrospinning

**DOI:** 10.1038/s41598-020-75906-9

**Published:** 2020-11-02

**Authors:** Gun Jin Kim, Kyu Oh Kim

**Affiliations:** Department of Fiber-System Engineering, Dankook University, 152, Jookjeon-ro, Suji-gu, Yongin-si, Gyeonggi-do 448-701 Republic of Korea

**Keywords:** Health care, Materials science

## Abstract

Micro- and nanofiber (NF) hydrogels fabricated by electrospinning to typically exhibit outstanding high porosity and specific surface area under hydrated conditions. However, the high crystallinity of NFs limits the achievement of transparency via electrospinning. Transparent poly(vinyl alcohol)/β-cyclodextrin polymer NF hydrogels contacted with reverse iontophoresis electrodes were prepared for the development of a non-invasive continuous monitoring biosensor platform of interstitial fluid glucose levels reaching ~ 1 mM. We designed the PVA/BTCA/β-CD/GOx/AuNPs NF hydrogels, which exhibit flexibility, biocompatibility, excellent absorptivity (DI water: 21.9 ± 1.9, PBS: 41.91 ± 3.4), good mechanical properties (dried: 12.1 MPa, wetted: 5.33 MPa), and high enzyme activity of 76.3%. Owing to the unique features of PVA/β-CD/GOx containing AuNPs NF hydrogels, such as high permeability to bio-substrates and rapid electron transfer, our biosensors demonstrate excellent sensing performance with a wide linear range, high sensitivity(47.2 μA mM^−1^), low sensing limit (0.01 mM), and rapid response time (< 15 s). The results indicate that the PVA/BTCA/β-CD/GOx/AuNPs NF hydrogel patch sensor can measure the glucose concentration in human serum and holds massive potential for future clinical applications.

## Introduction

In recent years, the design and development of wearable biosensors have garnered considerable attention owing to their promising potential in predictive analytics and personalized medical treatment. This is because of their ability to monitor the wearer’s health continuously and provide real-time health information, thereby facilitating remote monitoring^1^. Traditional clinical biosensors, which analyze urine and blood samples using standard analytical techniques are expensive, time-consuming, and not suited for the continuous measurement of target analytes. On the other hand, wearable biosensors are capable of a non-invasive analysis of biomarkers present in the saliva, tear, interstitial fluid (ISF), sweat, and blood. Furthermore, they can detect gas molecules contained in the exhaled breath.

Among the epidermal biofluids, ISF has a composition similar to blood in terms of essential small molecules, such as salts, proteins, glucose, and ethanol^2^. Over the years, researchers have used the ISF for the non-invasive diagnosis of metabolic disorders, therapy assessment, and organ failure evaluation. Guy et al. developed the reverse iontophoresis-based extraction of ISF from the skin^3,4^. In non-invasive glucose biosensors, the passage of low current (within 0.3 mA) across the skin draws the target analyte (i.e., glucose) to the epidermal surface. However, owing to the barrier properties of the skin, the concentration of electro-osmotically extracted glucose on the epidermal surface is very low. Therefore, compared to blood glucose monitoring, the current variations obtained for corresponding ISF glucose levels is small^5^. Thus, the development of a non-invasive biosensor capable of detecting low glucose levels in ISF with a low detection limit, high sensitivity, selectivity, faster response time, and good stability for long-term monitoring is deemed necessary. Hence, in this study, authors developed a flexible and stretchable nanofibrous membrane-based biosensor platform comprising electrodes modified with gold nanoparticles (AuNPs). AuNPs can help improve the sensing performance of non-invasive wearable biosensors.

Hydrogels are ideal synthetic materials that are preferred in a variety of biomimetic and biomedical applications owing to their similarities with the extracellular matrix, excellent biological performance, and inherent cellular interaction capability. However, engineered hydrogels exhibit low mechanical properties and different morphologies when compared with their natural counterparts. Additionally, several studies have investigated the preparation of porous hydrogels through the use of metal–organic frameworks^6^, gas formation^7^, salt leaching^8^, and freeze-drying method^9^. These studies have reported the limitations of these methods in controlling the pore size distribution. On the other hand, the pore size can easily be controlled using electrospinning, which can be used to synthesize micro- and nanofibers (NFs) with a high surface area to volume ratio based on materials, such as polymers, composites, and ceramics^10–12^. However, the opacity and white coloration resulting from their high crystallinity limit the use of NFs in various applications. Therefore, this study aims to fabricate a transparent electrospun hydrogel addressing these shortcomings. However, one major challenge in the application of porous and transparent hydrogel towards the development of non-invasive glucose biosensors is their inferior mechanical properties. On the other hand, the electrospun nanofibrous membrane owing to their high surface area can effectively immobilize enzymes compared to that of thin films^13^.

In a previous study, authors reported the fabrication of glucose-responsive poly(vinyl alcohol)/β-cyclodextrin (PVA/β-CD) hydrogels crosslinked with 1,2,3,4-butanetetracarboxylic acid (BTCA) using an eco-friendly synthesis procedure^14^. However, these hydrogel-based biosensors have low permeability, poor sensitivity, long reaction time, and unevenly distributed pores. Therefore, in this study, the development of highly conformable electrospun hydrogel NF-based biosensor platforms with high porosity and microscale porous morphology was investigated.

**Figure 1:**
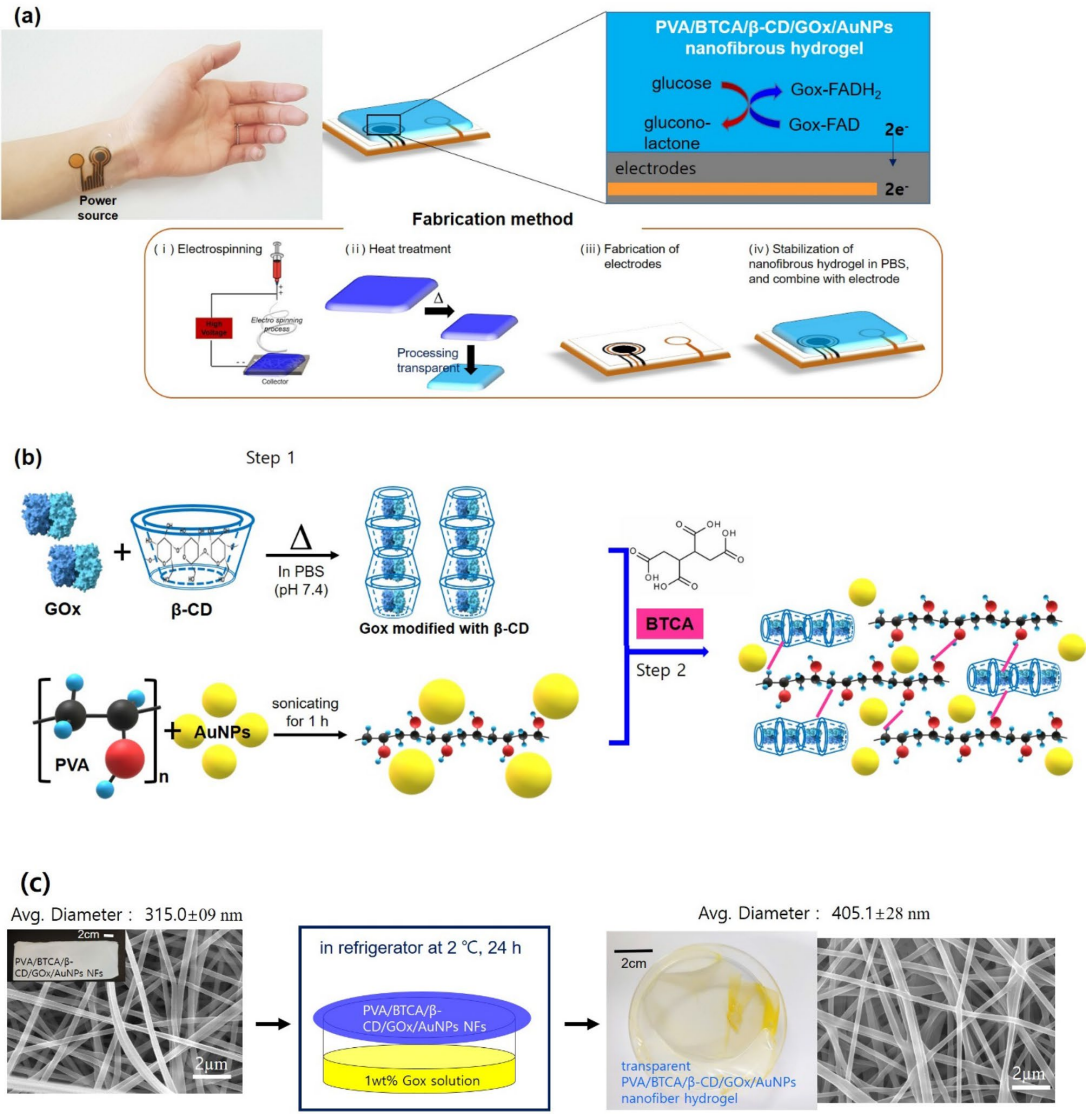
(**a**) Schematic illustration of the patch-type glucose sensor using PVA/BTCA/β-CD/GOx/AuNPs NF hydrogels on electrodes and the glucose-sensing mechanism for the noninvasive real-time monitoring of glucose in sweat. (**b**) Schematic illustration of the preparation of the PVA/BTCA/β-CD/GOx/AuNPs complex dope solution for electrospinning. (**c**) Process of transparent PVA/BTCA/β-CD/GOx/AuNPs nanofibrous hydrogels (containing optical and SEM images).

In this study, a skin patch sensor comprising electrospun hydrogel NFs were fabricated using two base polymers, namely, PVA and β-CD. PVA is known to exhibit excellent biological and physical properties^15^ as well as ease of hydrogel formation by chemical gelation using heat, γ-ray, or electron-beam irradiation^16,17^. Compared to acrylic polymer-based hydrogels, PVA exhibits negligible volume changes on water absorption^14^. CDs are cyclic oligomers composed of six, seven, or eight anhydrous glucopyranosyl units linked together by α-1,4-glycosidic bonds and are referred to as α-, β-, or γ-CD, respectively. β-CD can form inclusion complexes with many organic compounds and the formed cavities enable CDs to stabilize the host enzyme^14^. To prepare non-toxic hydrogels, formaldehyde-free BTCA was selected as the crosslinking agent. BTCA with four carboxylic acid groups can form ester bonds with hydroxyl groups. When polycarboxylic acids react with hydroxyl groups, a cyclic anhydride intermediate is formed due to the dehydration of two adjacent carboxyl groups at elevated temperatures. The anhydride intermediate reacts with hydroxyl groups and forms an ester linkage^18^. Esterification is achieved with heat treatment and salts of weak acids as catalysts^19^. The chemical structure of BTCA is shown in Fig. 1. Çay et al. demonstrated that BTCA-crosslinked PVA-hydrogel NFs are non-toxic, promote low weight loss, and retain their nanofibrous structure in the hot and cold water^20^. Au nanomaterials offer stable electrochemical properties, high catalytic activities, and biocompatibility. Such nanomaterials can maintain the activity of biological components while accelerating the electron transfer between immobilized enzymes and electrodes. Therefore, AuNPs are ideal materials for biosensor modification^21^.

Herein, authors report the fabrication of a transparent PVA/BTCA/β-CD/glucose oxidase (GOx) electrospun hydrogel incorporated with AuNP-based platforms capable of detecting glucose with high sensitivity. The bonding characteristics, absorption ratio, physical properties, and enzyme activity of the PVA/BTCA/β-CD/GOx/AuNP hydrogels were investigated. The electrochemical characteristics of thus fabricated sensor patch were tested using phosphate-buffered saline (PBS) and its glucose-sensing performance was evaluated. The lowest concentration of glucose that can be reliably detected from reverse iontophoresis-extracted ISF was determined. The results presented in this study contribute to both fundamental and clinical research as well as show high potential for continuous and non-invasive glucose monitoring from ISFs.

## Experimental

### Materials

The materials used include PVA (89% hydrolyzed, M_w_ = 85,000–124,000 g mol^−1^, Sigma-Aldrich), β-CD (beta-cyclodextrin, 99% purity, Mw = 1134.98 g mol^−1^, SAMCHUN), BTCA (1,2,3,4-Butanetetracarboxylic acid, 99% purity, Sigma-Aldrich), glucose oxidase enzyme from *Aspergillus niger* (GOx, Type X-S, lyophilized powder, ~ 100,000–250,000 units g^−1^, without added oxygen, Sigma-Aldrich), AuNPs (Gold nanoparticles, 50 nm diameter, stabilized suspension in 0.1 mM PBS, Sigma-Aldrich), and PBS (phosphate buffer saline, pH 7.4, Tech&Innovation). Other chemicals used, such as KH_2_PO_4_ and HCl, were of analytical grades. Double-distilled water was used throughout this study.

### Preparation of the β-CD/GOx solution

The 0.05 M of GOx solution, GOx (0.4 g, 75,000 units) was added to 5 mL of pH 7.4 PBS, stirred at 200 rpm for 2 h, and stored again at − 20 °C to maintain GOx activity. After thawing at room temperature, the mixture was stirred at 200 rpm for 30 min (F–T cycle). This process was repeated twice. β-CD was added to the prepared GOx solution, and the mixture was stirred at 70 °C for 1 h. The F–T cycle was performed thrice in this solution to form an inclusion compound between the β-CD.

### Preparation of the PVA/β-CD/AuNPs hydrogel NFs

PVA was added to distilled water to a concentration of 10% (w/v), stirred at 70 °C and 150 rpm for 3 h, and then stirred at room temperature overnight. Three milliliters of the AuNPs solution was added to 12 mL of the PVA solution, stirred for 30 min, and ultrasonically treated to enhance the dispersibility. The PVA/AuNPs solution and 5 mL of the β-CD/GOx solution were mixed and stirred at 70 °C and 200 rpm for 1 h. The stirred solution was cooled at room temperature, and then, 0.2 g of BTCA was added and further stirred for 1 h. The prepared solution was placed in a syringe and combined with a 21G metal needle. The syringe was fixed to a syringe pump such that the tip and collector distance (TCD) was 20 cm, and the aluminum foil was electrospun at a release rate of 0.6 mL h^−1^ and a voltage of 15 kV. The formed membrane was heated in a 110 °C convection oven for 6 h to be crosslinked. At this time, the carboxylic acid group of BTCA reacts with the hydroxyl group of PVA and β-CD to cause the esterification reaction. The crosslinked samples were stored in a freezer. For transparent PVA/BTCA/β-CD/GOx/AuNPs hydrogel NFs, the prepared membranes were placed on a 10 M GOx aqueous solution in a petri dish, covered, and stored in a freezer overnight at 0–2 °C, as shown in Fig. 1c.

### Field emission scanning electron microscopy

Field emission scanning electron microscopy (FE-SEM) was used to observe the morphology of each sample. At this time, the sample was analyzed in a sufficiently dried state. Each sample was coated with platinum at 20 mA for 150 s and captured using a Hitachi S-4700 device under vacuum conditions of 20 kV, Mag × 1.00 k, and WD 14.2 mm. The average fiber diameter of the fibers was measured using an image analysis software (Image J, National Institute of Health, USA) and calculated for 100 fibers that were randomly selected from the FE-SEM images of the scaffold.

### X-ray photoelectron spectroscopy

The crystal structure of the nanofibrous hydrogels was examined by X-ray analysis. X-ray diffractograms were obtained by two theta (2θ) scanning with the PHI 5000 VersaProbe (Ulvac-PHI, Japan) using monochromatized AlKα X-ray with a spatial resolution of 10 μm and 0.48 eV resolution conditions.

### Fourier-transform infrared spectroscopy

Fourier-transform infrared spectroscopy (FT-IR, A PerkinElmer Spectrum II) was used to collect spectra from 4000 to 450 cm^−1^ through the attenuated total reflectance (ATR) mode, to confirm that the carboxylic acid groups of BTCA and the hydroxyl groups of PVA and β-CD were bound by esterification and the amide group of GOx. The ρ_*Β*_ and ρ_*F*_ values of the nanofibers were calculated using Eqs. (1) and (2), respectively:

1 $${\rho }_{B} = \frac{4{m}_{1}}{{D}^{2}t}$$

2 $${\rho }_{F} = \frac{{m}_{1}}{{m}_{1}-{m}_{2}}{\rho }_{H}$$

where ${m}_{1}$ and ${m}_{2}$ are the masses of the nanofibers in air and n-hexane after a brief vacuum period, respectively. D and t are the diameter and thickness of the nanofibers, respectively.

### Enzymatic activity

After the heat treatment, the enzymatic activity of each sample was analyzed using a UV–visible instrument (UV/Vis Spectrometer, Lambda 25) using a glucose oxidase activity kit (Aldrich). GOx activity was determined by a coupled-enzyme assay, wherein the GOx oxidized d-glucose, resulting in the production of hydrogen peroxide (H_2_O_2_) that reacted with a probe, generating a colorimetric (570 nm)/fluorometric (λex = 535/λem = 587 nm) product, proportional to the GOx present. One unit of GOx is defined as the amount of enzyme that generates 1.0 μmol of H_2_O_2_ per min at 37 °C.

### Wide-angle X-ray scattering

Small-angle X-ray scattering (SAXS) analysis with a sampling volume of approximately 1 mm^3^ was performed to compare the crystallinity of the hydrogel NFs, while wide-angle X-ray scattering (WAXS) data were collected for approximately 18 h using a 2D wire detector (Bruker Nanostar) and analyzed using a combination of Fit2D software and customized analysis codes.

### Tensile properties

The tensile properties were analyzed using a universal testing machine (UTM) (Instron 3365 Duel-column electromechanical testing system). Tensile strength was measured using the ISO 1421 (tensile strength and elongation at break) method with the addition of pneumatic side action grips (2712-041) to Instron UTM. The strength of each sample during drying and the strength of each sample during wetting were measured to determine the relationship between the composition parameters of hydrogel NFs and mechanical properties and to determine whether the sample was suitable for use as a patch. Equation (3) was used to calculate the tensile strength as follows:

3 $$S=\frac{F}{A}$$

where S is the breaking strength (stress), F is the force that caused the failure, and A is the least cross-sectional area of the material. The mechanical properties of the hydrogel NFs showed a change in tensile strength depending on the constituent components.

### Absorptivity

The dried nanofibrous hydrogels were immersed in distilled water at room temperature. The weight of the samples was measured at regular intervals, and the maximum absorption was determined when the weight reached equilibrium. The water absorption (${W}_{a}$) of each hydrogel was calculated using Eq. (4):

4 $${W}_{a}=({W}_{e}-{W}_{0})/{W}_{0}$$

where ${W}_{e}$ is the weight of the hydrogel at the absorption equilibrium and ${W}_{0}$ is the first dry weight of the hydrogel.

### Cytotoxicity

The cytotoxicity of each sample on the viability of NIH 3T3 mouse embryonic fibroblast (purchased from the Korean Cell Line Bank) was measured using a WST-1 assay as a patch-type biosensor^22^. Each sample was immersed in 70% ethanol for 30 min and dried under sterile conditions. The samples were then exposed to UV light for 1 h and incubated in Dulbecco's Modified Eagle Medium (DMEM) for 24 h prior to cell seeding onto wells of 96‐well tissue culture plates. Cells were further seeded on nanofibrous hydrogels at a density of 1.0 × 10^4^ cells/well and incubated with 500 µL of DMEM supplemented with 10% FBS at 37 °C, 5% CO_2_ and 95% humidity. After incubation for 1 day, cells were incubated with the WST-1 labeled mixture for an additional 3 h. The absorbance of the WST-1 labeled mixture was measured at 450 nm using a microplate reader (VT, Bio-Tek Instruments, USA). The absorbance at 650 nm was used as a reference. Three different wells were measured for each culture condition, respectively (n = 3). All methods were carried out in accordance with relevant guidelines and regulations.

### Cyclic voltammetry

Cyclic voltage current method (CV) analysis using Prussian Blue (Engain, Inc.), Ag / AgCl and carbon coated with carbon (Netherlands is a 3-electrode system from CompactStat from Bium Technologies) is a sensor capable of electrochemical measurements Could. Reference and counter electrodes, respectively, under standard atmospheric temperature and pressure. Prior to the analysis, hydrogel NFs were fully swelled by immersion in pH 7.4 and 10 mM PBS for 10 min. The electrons emitted during the glucose oxidation process yielded a calibrated current to measure low glucose concentrations at 0.1–0.5 mM.

### Chronoamperometry

The amperometric response of the biocomposite to hydrogel NFs was recorded under steady-state conditions in stirred PBS (pH 7.4) by applying the desired potential to the electrode. The background current was collapsed to a steady state value before adding an aliquot of the stock glucose solution.

## Results and discussion

We successfully fabricated a thin and transparent PVA/BTCA/β-CD/AuNPs NF hydrogel with immobilized GOx via electrospinning, as shown in Fig. 1. For stabilization to the GOx enzyme, we encapsulated GOx from β-CD cavity by noncovalent host–guest inclusion complexes (Fig. 1b). The biocompatible synthetic polymer, PVA solution uniformly dispersed the AuNPs without aggregation. The PVA NFs containing GOx/β-CD inclusion complexes with a crosslinking agent (BTCA) and AuNPs were electrospun from the aqueous solution mixture. For comparison, PVA and PVA/GOx NFs without β-CD and AuNPs were also electrospun. After the electrospinning process, we treated the heat setting for crosslinking and then conducted enzyme vapor treatment overnight to form the transparent NF hydrogel at 2 °C. After treatment, we could observe the changing morphology and diameter (before: 315.0 ± 09 nm, after: 405.1 ± 28 nm) of the fiber through the SEM image, as shown in Fig. 1c. The PVA/BTCA/β-CD/GOx/AuNPs NFs hydrogel showed a relatively higher cell adhesion ratio and excellent interfacial biocompatibility than the other hydrogels presented in Supporting Data 1. Given that it did not irritate the skin, it was confirmed that this particular hydrogel could be satisfactorily used on the human body as patch biosensors.

**Figure 2:**
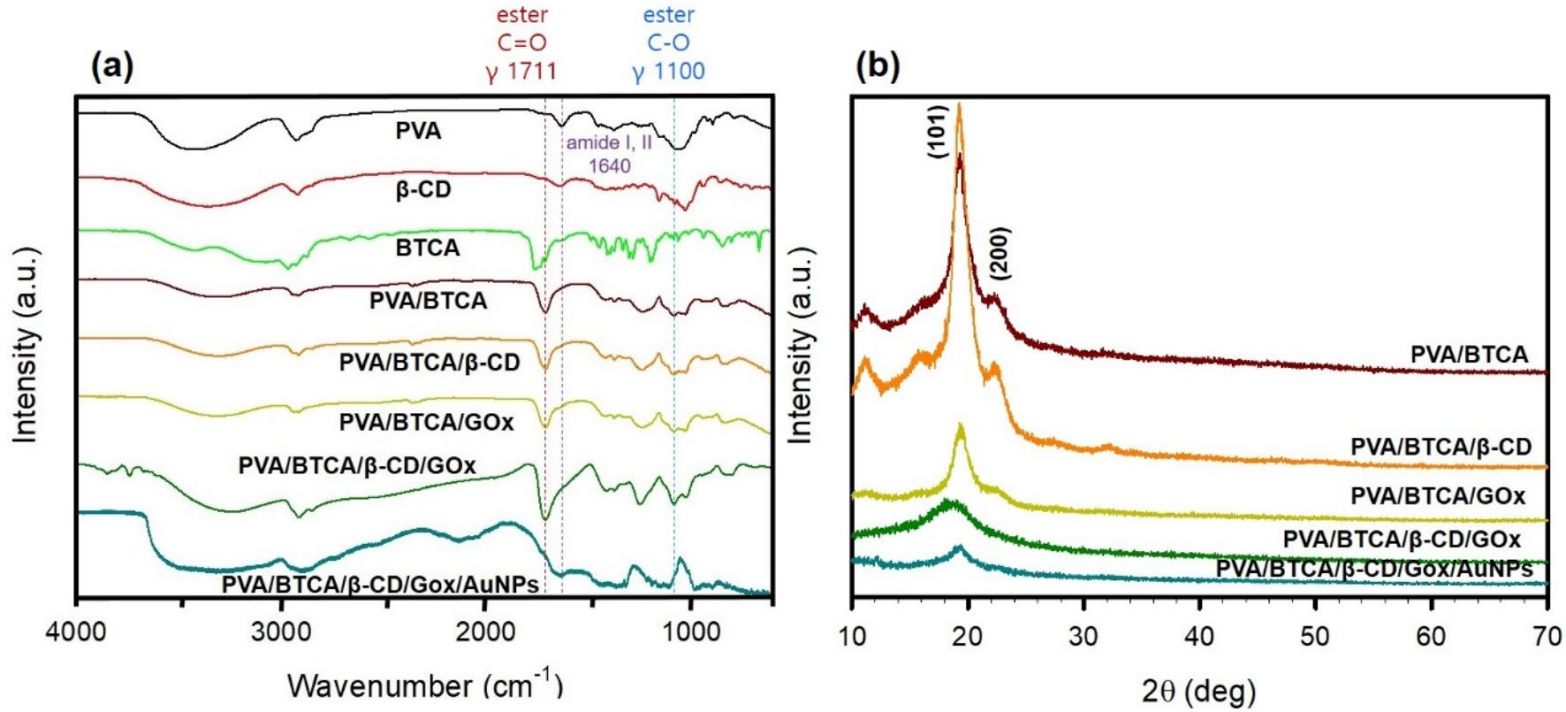
(**a**) FT-IR spectra and (**b**) XRD of PVA, β-CD, BTCA, PVA/BTCA, PVA/BTCA/β-CD, PVA/BTCA/GOx, PVA/BTCA/β-CD/GOx, and PVA/BTCA/β-CD/GOx/AuNPs NFs. The stretching and bending modes of different functional groups are indicated by γ and δ, respectively.

FT-IR spectra can provide valuable information about the esterification between the hydroxyl group and the carboxylic acid group on PVA/BTCA/β-CD/GOx/AuNPs NFs. The FT-IR spectra of (a) PVA, (b) β-CD, (c) BTCA, (d) PVA/BTCA, (e) PVA/BTCA/β-CD, (f) PVA/BTCA/GOx, (g) PVA/BTCA/β-CD/GOx, and (h) PVA/BTCA/β-CD/GOx/AuNPs are shown in Fig. 2a. The major peaks from the spectra of PVA include –OH broad stretching peak at 3400 cm^−1^, –CH stretching from alkyl groups at 2930 cm^−1^, –C–C stretching at 1630 cm^−1^, and –C–O stretching of alcohols at 1071 cm^−1^. β-CD shows –OH stretching at 3360 cm^−1^, –CH stretching from alkyl groups at 2910 cm^−1^, –C–C stretching at 1640 cm^−1^, and alcohol –C–O stretching at 1028 cm^−1^. BTCA shows –OH stretching at 3430 cm^−1^, –CH stretching at 2970 cm^−1^, carboxylic acid C=O stretching at 1720 cm^−1^, and –C–O stretching from alcohol and carboxylic acid at 1049 cm^−1^ and 1200 cm^−1^, respectively. When BTCA, containing PVA NF membranes, was subjected to heat treatment, an esterification reaction was expected to form between the hydroxyl groups of PVA and the carboxyl groups of BTCA. New vibration bands were observed for PVA/BTCA NFs at approximately 1712 cm^−1^, which can be attributed to the formation of ester C=O groups. Additionally, a stronger double peak was observed at 1243 cm^−1^ and 1100 cm^−1^, which could be assigned to the C–O stretching of esters. NFs contained with GOx was observed that the position of the amide I band in the enzyme estimated to be shifted from 1641 cm^−1^ to 1645 cm^−1^ that overlapped from C=O peak of PVA, whereas the amide II band at 1548 cm^−1^ in native GOx almost completely disappeared after the manufacturing NFs. Specially The PVA/BTCA/β-CD/GOx/AuNPs NFs has appeared the amide II peaks were observed at 1550 cm^−1^. These peaks of the sample containing AuNPs are indicative of the absence of a stronger bend. Enzyme comprise a specific sequence of their basic units, the amino acids whose arrangement into pleated sheets or α-helices accounts for their complex structures and biological activity. It has been reported that the free and exposed thiol groups in cysteine of enzymes have the capability for the reduction of Au metal salts to nanoparticles and provide excellent long-term stability because of the highest binding thiols ligand to the surface of Au metals^23,24^.

The influence of β-CD, GOx, AuNPs on the crystalline structure of crosslinked PVA was further studied by X-ray diffraction, as shown in Fig. 2b. PVA, as a semi-crystalline polymer, exhibits five typical diffraction peaks in the angular range of 10°–50°, diffraction peak of plane (100) at 11.5°, (101) at 19.5°, (101) at 20.1°, (200) at 23.0°, and a compound peak of crystalline planes of (111), (111), (210), and (210) at around 40.5°^25^. In Fig. 2b, it is shown an intense peak 19.5°, 23.0° relevant to PVA. As revealed, all these diffraction peaks for PVA/BTCA/β-CD/GOx/AuNPs NFs are weaker in intensity compared to PVA/BTCA, and the intensity continuously declines with increasing addition agent content, suggesting that the crystallization behavior of PVA chains is remarkably hindered. The additive values of the crystallinity degree were determined assuming absence of interactions between components. In general, the (101) diffraction is associated with the intermolecular interference among PVA chains and will provide useful information about the feature of hydrogen bonds. The enlarged (101) interplanar spacing suggests the weakened self-hydrogen bonding between PVA chains by generating the hydrogen bonding with β-CD, GOx molecules. Thus, the reduced degree in crystallization is indicating changes in blends morphology and interactions between components.

**Figure 3:**
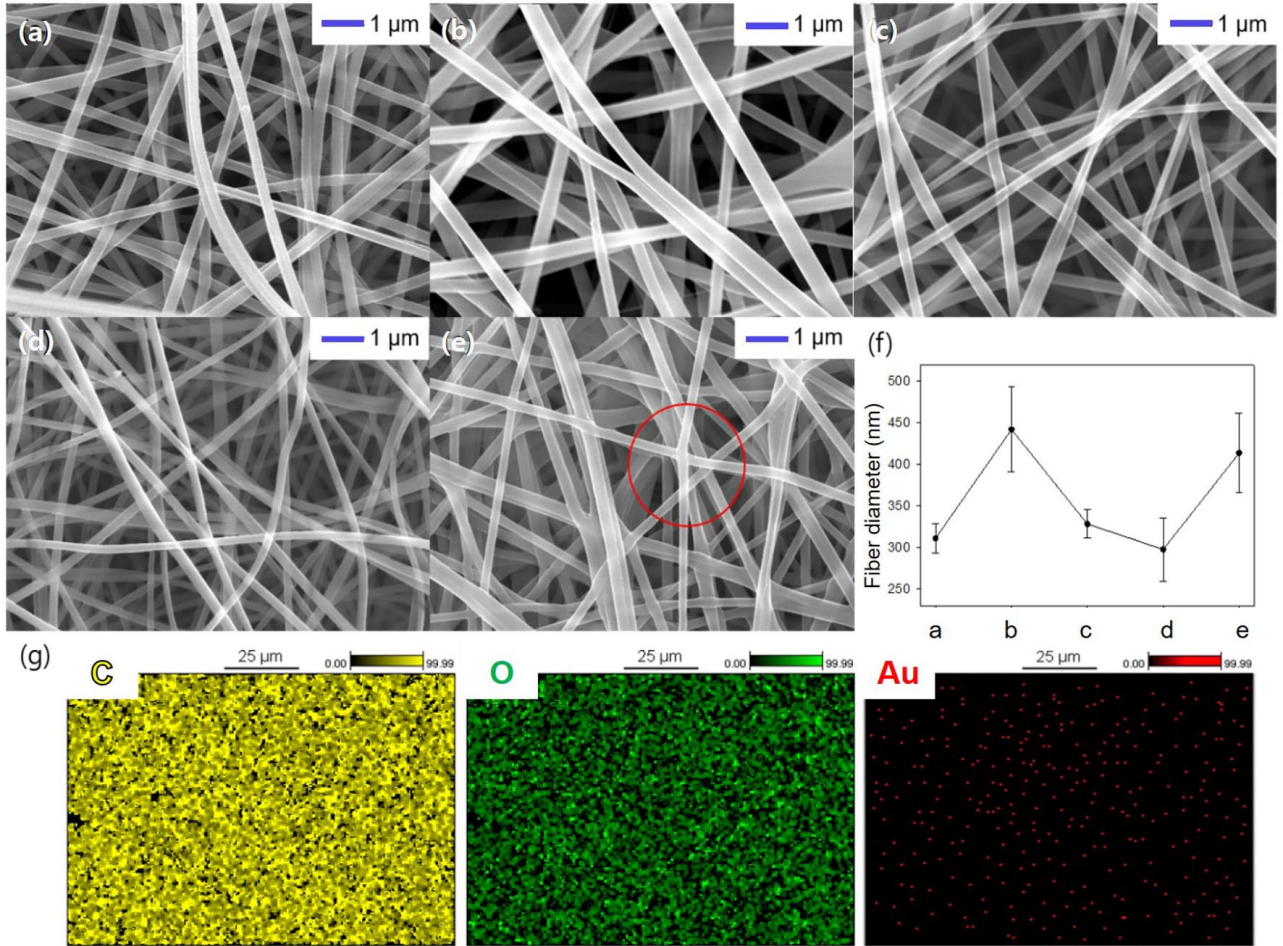
SEM micrographs of (**a**) PVA/BTCA, (**b**) PVA/BTCA/β-CD, (**c**) PVA/BTCA/GOx, (**d**) PVA/BTCA/β-CD/GOx, (**e**) PVA/BTCA/β-CD/GOx/AuNPs hydrogel NFs, (**f**) average diameters of each sample, and (**g**) elemental mapping of carbon (yellow), oxygen (green), and gold (red) elemental dispersions in the PVA/BTCA/β-CD/GOx/AuNPs NFs.

Long continuous PVA/β-CD/AuNPs NFs were fabricated by electrospinning in this study. The resulting PVA/BTCA, PVA/BTCA/β-CD, PVA/BTCA/GOx, PVA/BTCA/β-CD/GOx, and PVA/BTCA/β-CD/GOx/AuNPs NFs were collected as a nonwoven mat and had a smooth surface morphology, as shown in Fig. 3a–e. No defects, such as beads, pores, or ribbons, were found in/on the NFs. We was observed that the crossed fibers adhered to each other at Fig. 3(e, marked with a red circle), without changing the shape and morphology. The ultrafine PVA/β-CD/AuNPs NFs displayed a centralized diameter distribution ranging from 298 to 442 nm (Fig. 3f). We observed that the fiber diameter decreased with the inclusion of the GOx because a polar ligand in the enzyme conduct the electrical conductivity and interrupts chain entanglement. The elemental mapping of PVA/BTCA/β-CD/GOx/AuNPs NFs is shown in Fig. 3g. The presence of Au was confirmed by elemental mapping (Fig. 3g), where the homogeneous distribution of elemental Au in the PVA/BTCA/β-CD/GOx membranes confirmed the successful immobilization of AuNPs.

**Figure 4:**
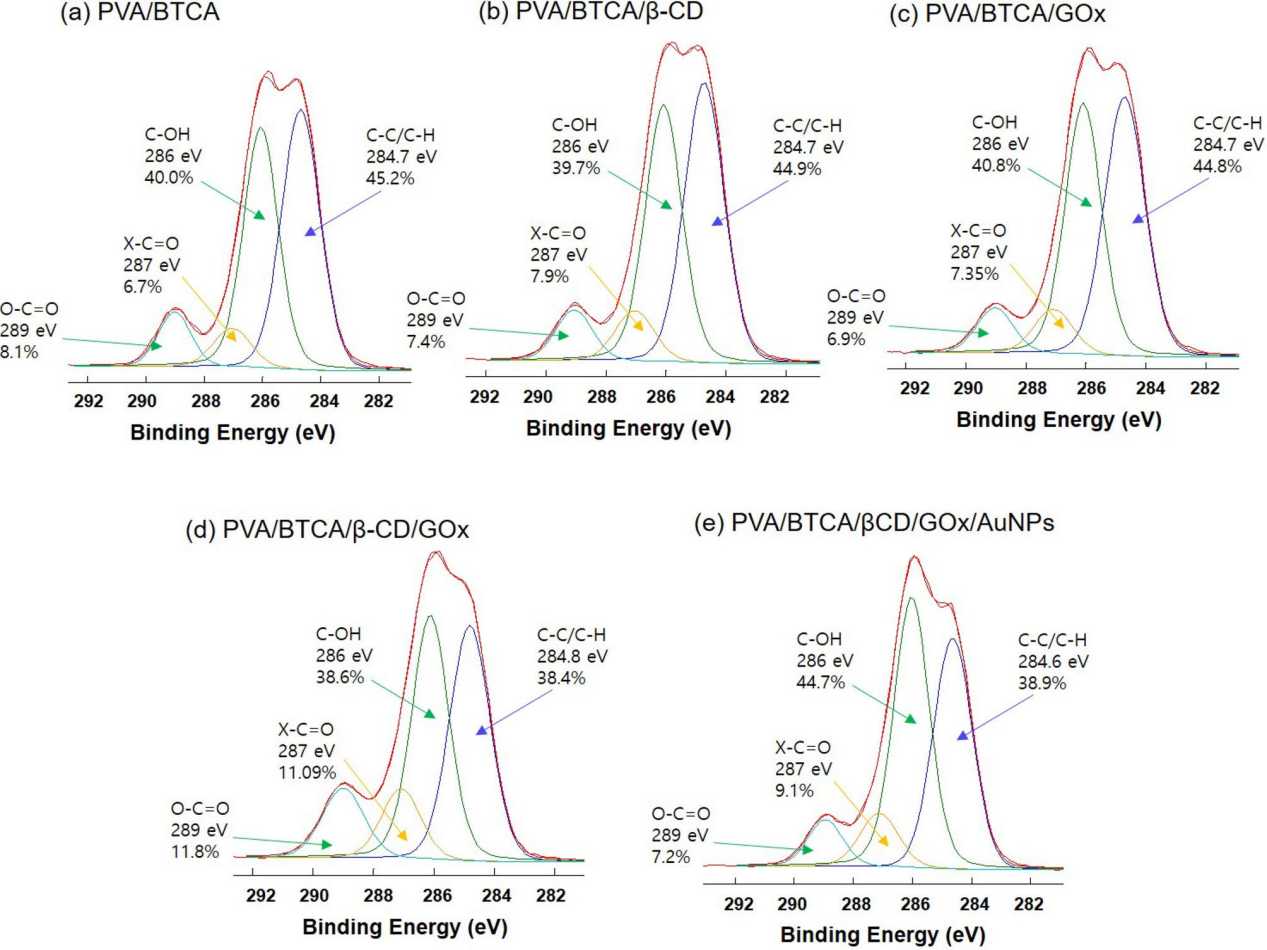
XPS spectrum of hydrogel NFs for the confirmation of the formation of inclusion compounds of secondary binding energy spectrum of carbon of (**a**) PVA/BTCA, (**b**) PVA/BTCA/β-CD, (**c**) PVA/BTCA/GOx, (**d**) PVA/BTCA/β-CD/GOx, and (**e**) PVA/BTCA/β-CD/GOx/AuNPs.

The survey XPS spectra in Fig. 4 show, for each PVA/BTCA NF, an expected surface with intense C1s signals. XPS is a quantitative spectroscopic technique that measures the elemental composition, chemical state, and electronic state of the elements that exist within a material and provides the chemical bonding information of the material. Figure 4 shows the C1s XPS spectra of the PVA/BTCA, (b) PVA/BTCA/β-CD, (c) PVA/BTCA/GOx, (d) PVA/BTCA/β-CD/GOx, and (e) PVA/BTCA/β-CD/GOx/AuNPs. As shown, there are four kinds of carbon atoms in different functional groups, such as ring carbons (C–C) at ~ 284.6–284.8 eV, carbons in phenolic hydroxyl groups (C–OH/C–N) at 286.0 eV, carbons in epoxy/ether (C–O–C) at 287.0 eV, and carboxylic carbons (O–C=O) at 289.0 eV. The peaks of the C–OH/C–N of (d) and (e) are obviously higher than those of (a), (b), and (c), which indicates that these glucose oxidases in (d) and (e) are stably immobilized into the β-CD cavities^26^. In particular, the NF-containing AuNPs were more evidently verified. It can be shown that this result corresponds to the electrochemical sensing data in Fig. 7.

It is apparent that the mechanical properties of all the hydrated samples were remarkably lower than those of the dried samples. Dried and hydrated samples showed differences in the behavior of breaking.

**Figure 5:**
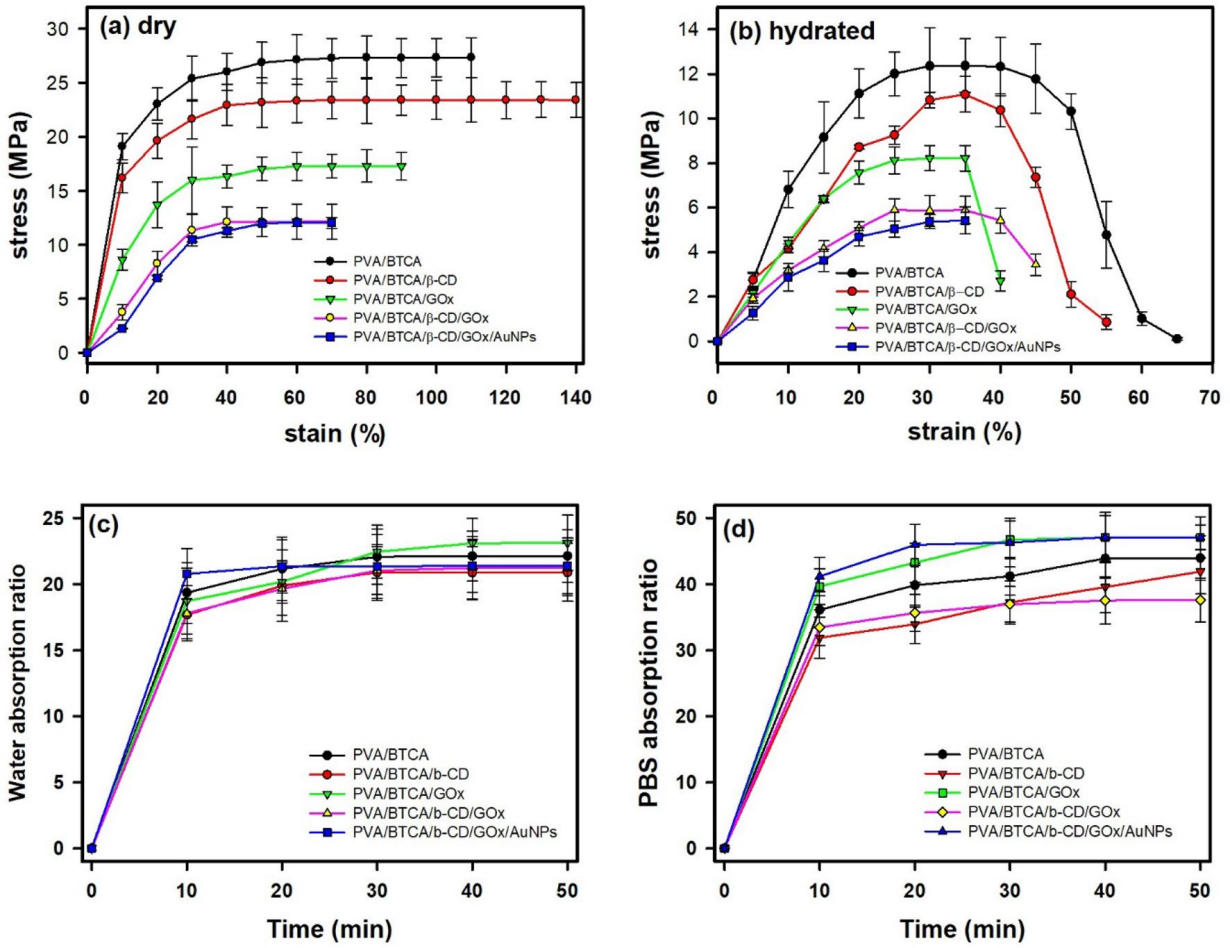
Tensile stress–strain curves for PVA/BTCA, PVA/BTCA/β-CD, PVA/BTCA/GOx, PVA/BTCA/β-CD/GOx, and PVA/BTCA/β-CD/GOx/AuNPs hydrogel NF (**a**) in a dry state, (**b**) in a hydrated state, (**c**) absorption ratio for distilled water, and (**d**) absorption ratio for PBS solution.

As shown in Fig. 5a, the stress–strain (S–S) characteristics of PVA/BTCA, PVA/BTCA/β-CD, PVA/BTCA/GOx, PVA/BTCA/β-CD/GOx, and PVA/BTCA/β-CD/GOx/AuNPs NFs were measured to determine the mechanical properties of the hydrogel NFs in a dry and hydrated state for use as patch-type sensors. The dried NF hydrogels demonstrate typical nonlinear S–S characteristics, behaving like a viscoelastic solid with minimal hysteresis under repeated cyclic loading. The stress of PVA/BTCA, PVA/BTCA/β-CD, PVA/BTCA/GOx, PVA/BTCA/β-CD/GOx, and PVA/BTCA/β-CD/GOx/AuNPs hydrogel NFs in a dry state was 27.3 ± 1.8, 23.4 ± 1.7, 17.2 ± 1.1, 12.3 ± 1.6, and 12.0 ± 0.5 MPa, while in a hydrated state (Fig. 5b), they were 12.3, 11.1, 8.1, 5.9, and 5.3 MPa. The difference in behavior was approximately 5 MPa depending on the presence of GOx in the wet state. If materials are composited into the PVA polymer chain, the physical properties fall as a whole owing to the collapse of the crystal structure of PVA. To increase the mechanical properties of the PVA/BTCA/β-CD/GOx/AuNPs, it is possible to control the amount of crosslinking of BTCA^13^. The s–s curves did not show a similar curve shape for the dried state and hydrated state, and the stress and strain of the NF hydrogels in the hydrated state were lower than those of the dried NF hydrogels. The difference between them was that hydrated hydrogels showed necking and breaking up at the fracture point. Unlike in the dried state, a break occurs in 1 s on the ultimate stress point without the necking zone. However, it has sufficient mechanical properties to be used as a biosensor and for handling.

The absorption ratios of PVA/BTCA, PVA/BTCA/β-CD, PVA/BTCA/GOx, PVA/BTCA/β-CD/GOx, and PVA/BTCA/β-CD/GOx/AuNPs hydrogel NFs are shown in Fig. 5c for distilled water and Fig. 5d for PBS solution. The highest water absorption ratio of all samples was approximately 21.9 ± 1.9 at 20 min. The highest PBS absorption ratio of PVA/BTCA, PVA/BTCA/β-CD, PVA/BTCA/GOx, PVA/BTCA/β-CD/GOx, and PVA/BTCA/β-CD/GOx/AuNPs hydrogel NFs are 31.9 ± 3.1, 34.0 ± 2.9, 37.23 ± 3.3, 39.56 ± 3.9, and 41.91 ± 3.4, respectively. PVA hydrogels have been known to possess a high water content^11,27^. All of the samples based on PVA hydrogel NFs quickly reached the highest absorption ratio within 10 min. The PBS absorption ratio was higher than that of water. Generally, the hydrophobicity of β-CD indicates a lower water absorptivity than the PVA only hydrogel^28^. However, the GOx-loaded PVA/β-CD hydrogel shows decreased hydrophobicity because the hydrophobic portion of GOx is physically entangled within the hydrophobic cavity of the β-CD with the hydrophilic GOx segment remaining on the molecular surface. PVA/BTCA/β-CD/GOx/AuNPs hydrogel NFs showed optimal properties with excellent water absorption.

**Figure 6:**
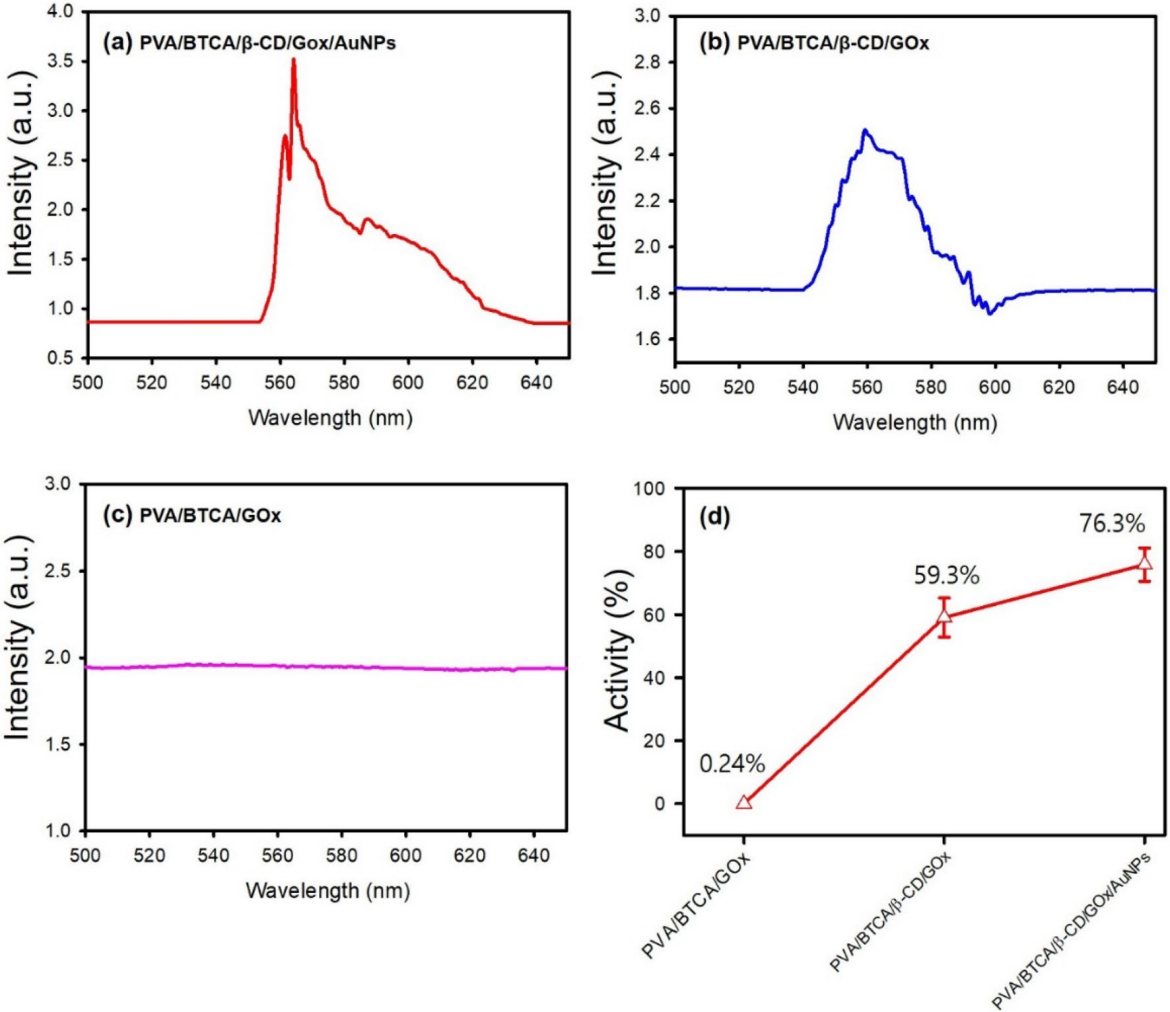
Analysis of enzymatic activity of (**a**) PVA/BTCA/β-CD/GOx/AuNPs, (**b**) PVA/BTCA/β-CD/GOx, and (**c**) PVA/BTCA/GOx hydrogel NFs after heat treatment using a glucose oxidase activity kit and UV–Vis spectroscopy. The colorimetric (535 – 570 nm) product of GOx produced by the GOx activity kit via d-glucose oxidation and reaction with the probe to produce hydrogen peroxide (H_2_O_2_), (**d**) absorbance at a wavelength of 570 nm, which is the maximum absorbance of each sample.

To confirm the stability of the enzyme in PVA/BTCA/β-CD/GOx/AuNPs hydrogel NFs, the enzyme activity was assayed using the active kit and the UV–Vis instrument. The enzymatic activities of PVA/BTCA/GOx, PVA/BTCA/β-CD/GOx, and PVA/BTCA/β-CD/GOx/AuNPs hydrogel NFs after heat treatment are shown in Fig. 6. Nano hydrogel membranes are manufactured through a harsh environment, such as the extremely high voltage of the electrospinning process and heat treatment (for 6 h at 110 °C) for crosslinking. Therefore, PVA/BTCA/GOx causes 0.24% enzyme activity and most of the enzymes are denatured and stop working. The enzymatic activity of the PVA/BTCA/β-CD/GOx hydrogel showed that the formation of the GOx-β-CD inclusion compound improved the stability of the enzyme and showed that the enzyme could be activated even after electrospinning and heat treatment. The presence of AuNPs hydrogel NFs appeared extremely to increase enzyme activity by 76.3% because of the higher affinity of the linear S–Au–S complex^21^. These results indicate that PVA/BTCA/β-CD/GOx/AuNPs hydrogel NFs are suitable for use as biosensor patch materials.

**Figure 7:**
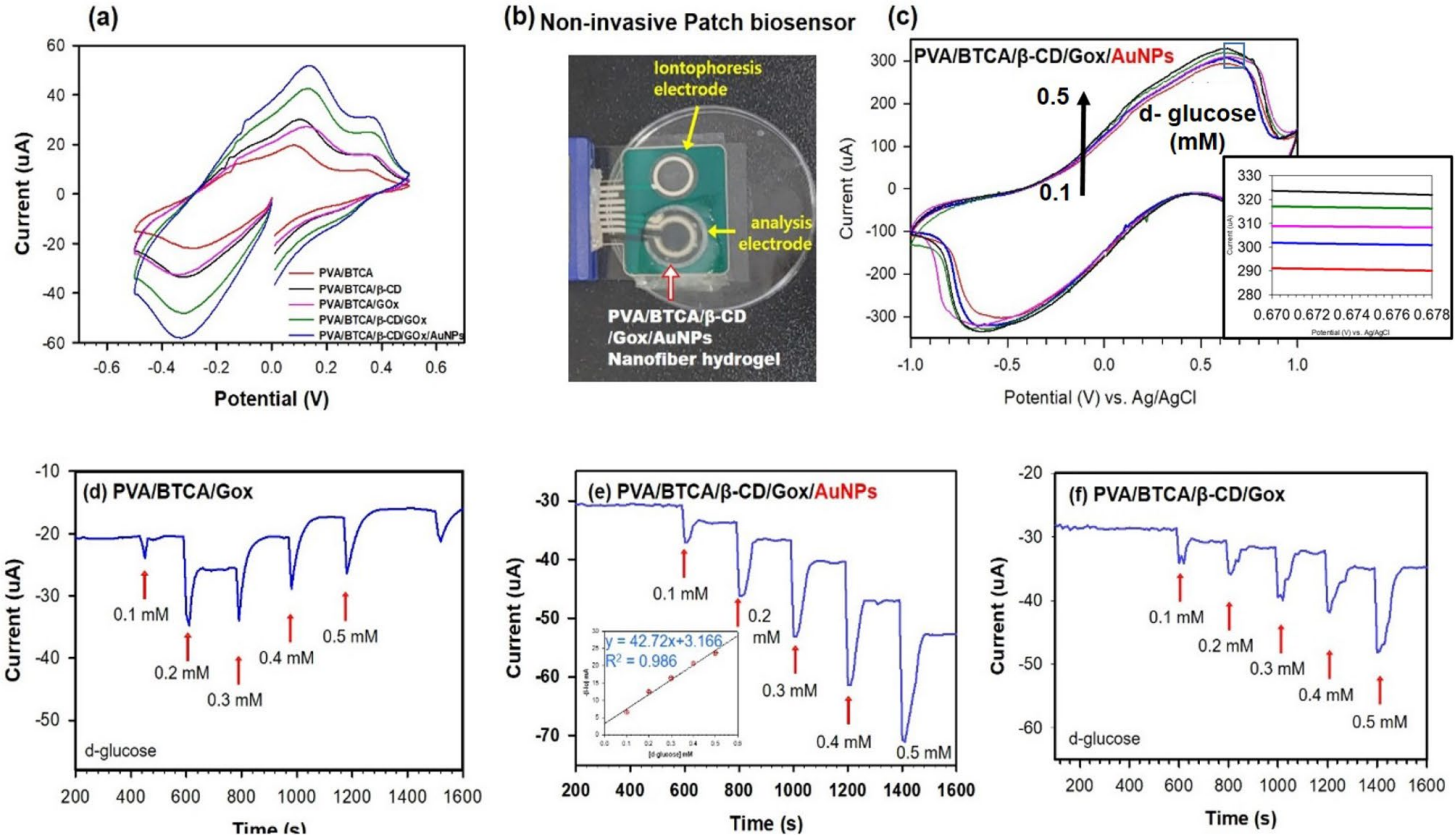
(**a**) CVs of PVA/BTCA, PVA/BTCA/β-CD, PVA/BTCA/GOx, PVA/BTCA/β-CD/GOx, and PVA/BTCA/β-CD/GOx/AuNPs hydrogel NFs hydrated by PBS (pH 7.4); Scan rate: 0.1 Vs^−1^; (**b**) three-electrode measurement of patch-type PVA/BTCA/β-CD/GOx/AuNPs hydrogel NFs hydrated (**c**) CVs of PVA/BTCA/β-CD/GOx/AuNPs at varied absorbed d-glucose concentrations (0.1–0.5 mM) at pH 7.4 and 25 °C; Scan rate: 0.1 V s^−1^ and amperometric responses to successive addition of d-glucose (0.1–0.5 mM) at − 0.2 V (vs. Ag/AgCl) on the PVA/BTCA/GOx (**d**), PVA/BTCA/β-CD/GOx/AuNPs (**e**), PVA/BTCA/β-CD/GOx (**f**). The inset (**e**) shows the calibration plot of the steady-state current as a function of d-glucose concentration.

The electrochemistry of GOx-loaded hydrogel NFs hydrated with PBS (pH 7.4) was studied by cyclic voltammetry. Figure 7a shows the CVs of PVA/BTCA, PVA/BTCA/β-CD, PVA/BTCA/GOx, PVA/BTCA/β-CD/GOx, and PVA/BTCA/β-CD/GOx/AuNPs NF hydrogel hydrated with PBS (pH 7.4) at a scan rate of 0.1 V s^−1^. The background current of the PVA/BTCA/β-CD/GOx/AuNPs was evidently higher than that of the other hydrogels, which is ascribed to the improvement of electrical conductivity by AuNPs. A pair of well-defined and quasi-reversible redox peaks was observed at the CVs of PVA/BTCA/β-CD/GOx/AuNPs (Fig. 7c, black line) with the anodic peak potential (E_pa_) at 0.624 V and the cathodic peak potential (E_pc_) at − 0.637 V. The formal potential (E^0′^ = (E_pa_ + E_pc_)/2) of PVA/BTCA/β-CD/GOx/AuNPs was − 6.5 mV which is close to the electrode potential of GOD in previous reports^29,30^. The peak-to-peak separation (ΔE_p_) is approximately 13 mV Such a small Ep value reveals a fast and quasi-reversible electrontransfer process, revealing a fast electron transfer process among the hydrogels being studied^4^.

Figure 7c shows the CVs of PVA/BTCA/β-CD/GOx/AuNPs in PBS (10 mM, pH 7.4) with different glucose concentrations. As shown, the cathodic peak current decreased linearly with the increase in glucose concentration (0.1–0.5 mM). We conducted chronoamperometric measurements of the PVA/BTCA/GOx, PVA/BTCA/β-CD/GOx/AuNPs and PVA/BTCA/β-CD/GOx NF hydrogel biosensors as a function of glucose concentration at room temperature (Fig. 7d–f). The chronoamperometric current responses determine the difference in the sensing performance of each membrane. In particular, the PVA/BTCA/β-CD/GOx/AuNPs NF hydrogel sensor has excellent sensing ability. The PVA/BTCA/GOx hydrogel sensor has a significantly low measurement accuracy. This could be explained by the lower enzyme activity and stability of PVA/BTCA/β-CD/GOx were lower than those of PVA/BTCA/β-CD/GOx/AuNPs. We obtained the calibration plot of the steady-state current as a function of glucose concentration from 0.1 mM to 0.5 mM PVA/BTCA/β-CD/GOx/AuNPs (inset of Fig. 7e inset. This biosensor showed an increase in the standard deviation as the glucose concentration increased. The PVA/BTCA/β-CD/GOx/AuNPs NF biosensor achieved linearity with a high correlation coefficient (R^2^) of 0.98 and exhibited a fast response time of ˂ 15 s. The detection limit (DL) was estimated based on applying the signal-to-noise ratio equation (S/N = 3)^7^ (Eq. 5):

5 $$\mathrm{DL}=\frac{3s}{k}$$

where s is the standard deviation for the instrument (i.e., 1.30 × 10^–10^) and k is the slope of the i–c curve. A detection limit of 0.01 mM was obtained together with a linear range of 0–0.5 mM, with a sensitivity of 47.2 μA mM^−1^. Although present in ISF at a reasonable level, the extracted glucose was estimated to be present at a much lower level (12.5–125 mM) during each sampling period (15 min)^31^; hence, the detection range and sensitivity of the biosensor are adequate to determine the levels of extracted glucose.

In the market, the traditional glucose sensor is invasive, and the tested concentration is much higher than that of the sensor in this study. Overall, our research shows that the modification method is feasible, and it provides the possibility of being applied to other physiological fluids (such as subcutaneous tissue fluid, ISF, and saliva), where the concentration of glucose is lower than that in blood.

## Conclusion

An amperometric wearable glucose sensor, PVA/BTCA/β-CD/GOx/AuNPs NF hydrogel, was assembled by electrospinning using (PVA), BTCA (biocompatible crosslinker), and AuNPs with a modified β-CD–glucose oxidase enzyme (GOx) complex carrier. The glucose-responsive transparent PVA/BTCA/β-CD/GOx/AuNPs NF hydrogels had a biocompatible, thin and flexible, high absorbency (DI water: 21.9 ± 1.9, PBS: 41.91 ± 3.4), well-balanced mechanical properties (dried: 12.1 MPa, wetted: 5.33 MPa), and a high enzyme activity of 76.3%. The cyclic voltammetric result of the electrode shows a pair of well-defined and quasi-reversible redox peaks with a formal potential of − 6.5 mV and a peak to peak separation of 13 mV, revealing that the direct electron transfer between GOx and the electrode has been achieved. Moreover, the linear range for the glucose concentration was 0.1 mM to 0.5 mM with a sensitivity of 47.2 μA mM^−1^ and a detection limit of 0.01 mM and an R^2^ value approaching 1. Considering the facile and scalable processability of hydrogels, the proposed transparent PVA/BTCA/β-CD/GOx/AuNPs NF hydrogel-based biosensor platform has good potential in wearable healthcare monitoring systems, clinical diagnostics, and biomedical devices.

## Supplementary information

Supplementary Figure.
