# Supplementary material for: Novel glucose-responsive of the transparent nanofiber hydrogel patches as a wearable biosensor via electrospinning
Source: Sci Rep. 2020 Nov 2;10:18858. doi: 10.1038/s41598-020-75906-9 (PMC7608638; doi:10.1038/s41598-020-75906-9)
Supplement: Supplementary file 1 — Supplementary Figure. [file 41598_2020_75906_MOESM1_ESM.docx]

Supplementary information about the article

“Novel Glucose-responsive of the Transparent Nanofiber Hydrogel Patches as a Wearable Biosensor via Electrospinning”

Gun jin Kim^1^, Kyu Oh Kim^1^,*

^1^Department of Fiber-system Engineering, Dankook University

*Corresponding author with contact details:

• E-mail address: affablekim@gmail.com (K.O. KIM)

• Full postal address: 152, Jookjeon-ro, Suji-gu, Yongin-si, Gyeonggi-do 448-701, Republic of Korea

• Tel.: +82 31 8005 3561; Fax: +82 31 8005 3564


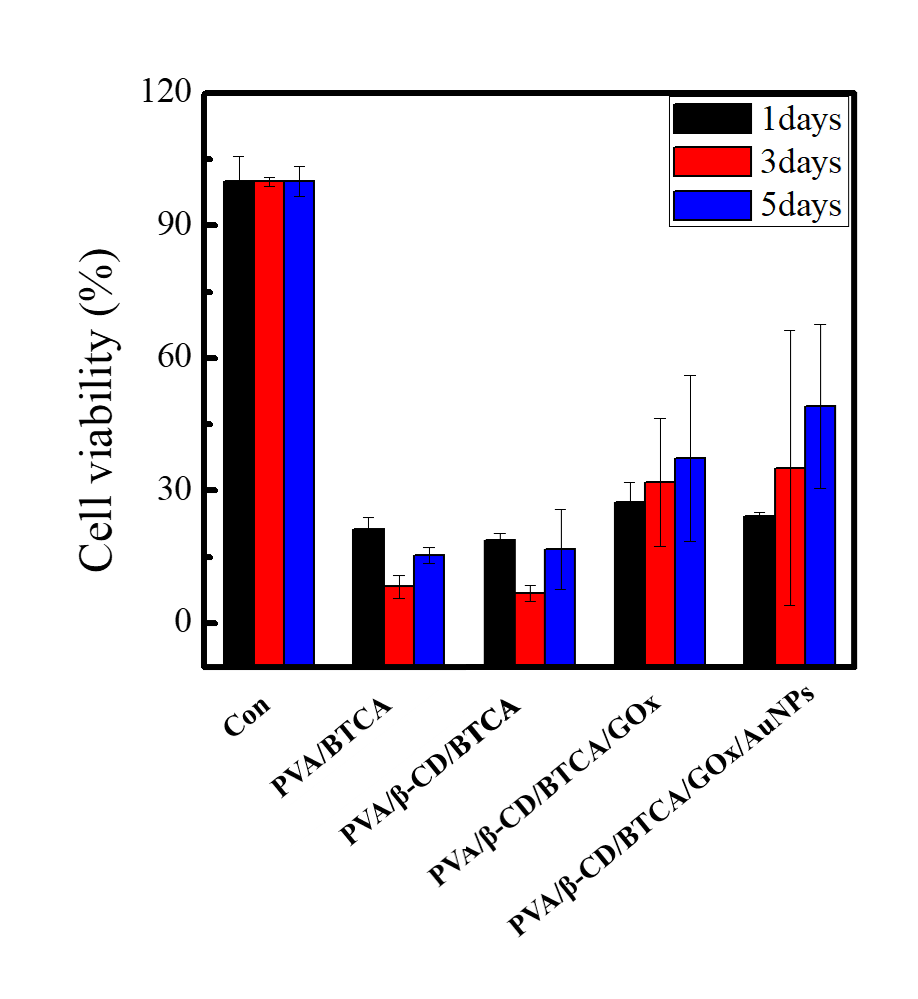


Figure S1: Cytotoxicity data of PVA/BTCA, PVA/BTCA/β-CD, PVA/BTCA/GOx, PVA/BTCA/β-CD/GOx, and PVA/BTCA/β-CD/GOx/AuNPs hydrogel NFs at 1 day(black), 3 days (red), and 5 days (blue).
